# Managing Security of Healthcare Data for a Modern Healthcare System

**DOI:** 10.3390/s23073612

**Published:** 2023-03-30

**Authors:** Abdulmohsen Almalawi, Asif Irshad Khan, Fawaz Alsolami, Yoosef B. Abushark, Ahmed S. Alfakeeh

**Affiliations:** 1 Computer Science Department, Faculty of Computing and Information Technology, King Abdulaziz University, Jeddah 21589, Saudi Arabia; 2 Department of Information Systems, Faculty of Computing and Information Technology, King Abdulaziz University, Jeddah 21589, Saudi Arabia

**Keywords:** smart hospital system, internet of things, security and privacy, optimization, artificial intelligence, healthcare data

## Abstract

The advent of Artificial Intelligence (AI) and the Internet of Things (IoT) have recently created previously unimaginable opportunities for boosting clinical and patient services, reducing costs and improving community health. Yet, a fundamental challenge that the modern healthcare management system faces is storing and securely transferring data. Therefore, this research proposes a novel Lionized remora optimization-based serpent (LRO-S) encryption method to encrypt sensitive data and reduce privacy breaches and cyber-attacks from unauthorized users and hackers. The LRO-S method is the combination of hybrid metaheuristic optimization and improved security algorithm. The fitness functions of lion and remora are combined to create a new algorithm for security key generation, which is provided to the serpent encryption algorithm. The LRO-S technique encrypts sensitive patient data before storing it in the cloud. The primary goal of this study is to improve the safety and adaptability of medical professionals’ access to cloud-based patient-sensitive data more securely. The experiment’s findings suggest that the secret keys generated are sufficiently random and one of a kind to provide adequate protection for the data stored in modern healthcare management systems. The proposed method minimizes the time needed to encrypt and decrypt data and improves privacy standards. This study found that the suggested technique outperformed previous techniques in terms of reducing execution time and is cost-effective.

## 1. Introduction

Technological advancements have made a modern method to improve human life quality possible [1]. The IoT is an innovative and developing paradigm gaining interest in several application sectors, including smart homes, smart environments, and personal and remote healthcare [2]. Research and technology researchers have identified and evaluated health data sources to learn more and solve health-related challenges [3]. Therefore, creating integrated healthcare technology can boost productivity and increase patient outcomes at every level of the medical system [4]. The world’s largest and fastest-growing industry is the healthcare sector. How healthcare is managed has changed over the past several years from a disease-centered approach to a patient-centered one [5] and a volume-based approach to a value-based strategy of healthcare delivery. The growing drive for patient-centered treatment and value-based healthcare delivery models is guided by the goals of raising public awareness of the excellence of healthcare and reducing costs [6].

By utilizing strong patient safety controls, widespread access to data, remote inpatient monitoring, quick intervention strategies, and decentralized electronic medical records, the creation of new IoT-based healthcare software applications can address some issues associated with conventional healthcare systems [7]. A system created to handle healthcare data is referred to as a medical information system (MIS). This includes the practical administration of a hospital or a system that supports the formulation of healthcare policy, as well as systems that gather, store, handle, and send a patient’s electronic medical record (EMR) [8]. These techniques can increase the quality of life for patients, boost cooperation, boost patient outcomes, lower costs, and boost the overall efficiency of e-healthcare services [9]. Systems that manage data linked to the operations of providers and healthcare groups are also included in the category of health information systems. These could be used in concert to impact research, better patient results, and improve policy and decision making. Because expenditures in extensive data analysis can be significant and create a demand for effective, affordable technology, using the cloud to study big data in healthcare stands to reason [10]. Security is a top priority because medical information systems frequently view, handle, or keep huge amounts of sensitive data. Since the equipment is typically attached to an internal network that is linked to the Internet, it is also susceptible to viruses from devices and other equipment carried into hospitals. Different kinds of malicious attacks can be caused by the attackers [11].

A form of malicious software known as ransomware stops you from reaching your device’s information, infrastructure, or networks and requests a ransom in exchange for their release. These assaults, which have been linked to issues with medical processes, disrupted patient treatment, according to more than half of ransomware victims. The probability of returning to care redirection following an assault was the greatest impact noted. If a hacker seizes control, they can instruct devices to provide false readings, deliver medication drug overdoses, or take other actions that jeopardize the health of patients [12]. Due to the substantial quantity of confidential data that healthcare organizations keep for patient treatment and activities, the sector is seen as a target-rich environment. Consequently, cybercriminals have shifted their focus from the banking industry and retail shops to healthcare facilities due to personal health information potential value being up to 50 times greater than finance data; it can be valuable to attackers. Hospitals are important infrastructure companies that keep, exchange, and use a lot of private information. To provide patients with vital medical treatment, healthcare centers also rely on a number of IoT devices and electronic medical records [13]. This particular combo appeals to cybercriminals as a prize deserving of a hefty ransom that will be rapidly paid. However, becoming a primary target involves more than just motivation and pressure. Hospitals are an excellent target for devastating malware assaults as a result of a number of current occurrences that have combined to create the perfect storm [14].

Identity theft is a major problem for cybercriminals, as it can lead to the theft of personal information such as insurance, names, policy numbers, birth dates, billing data, diagnosis codes, and bank and credit card information [15]. Fraudsters use data from healthcare organizations to create fake IDs, resell medical equipment, and file made-up claims with insurers. Many users are unaware that they have been compromised, leading to unexpected consequences and rampant medical card theft. Medical identity theft is the act of someone using confidential information such as a social security number, without permission, to make false claims to Medicare and other health insurers, which can waste government money and interfere with medical treatment [16]. These identity thefts are correlated to criminal forgery theft. The use of tools, procedures, and measures to defend against cyberattacks on networks, applications, gadgets, systems, and data is known as cyber security. Its objectives are to lower the danger of cyberattacks and safeguard against the unauthorized use of innovations, networks, and platforms [17].

Early in the COVID-19 pandemic, it was unclear how healthcare costs and use would alter globally. Although a pandemic may lead to higher health expenses, spending and use declined [18] due to other considerations. The cost of combating fraud and upholding rules is a factor. Expensive antivirus software must be obtained to shield private patient data from hackers [19]. Due to this, healthcare costs must increase to maintain patient and data security. AI and machine learning have revolutionized healthcare, particularly in medical specialties. The medical disciplines make significant use of computer systems with artificial intelligence, such as remote patient treatment, prescription transcription, enhancing doctor–patient contact, drug research and development from beginning to finish, and patient diagnosis [20]. Modern computer algorithms have recently attained accuracy levels that are comparable to those of human specialists in the field of medical sciences, despite the fact that computer systems frequently perform jobs more quickly than humans do. The goal of separating rhetoric from reality is discussing how AI is reshaping the field of medicine. AI can help healthcare organizations cut costs by deploying more sophisticated technology that is more accurate and well-suited to carry out particular functions [21]. Ensuring that the appropriate care and support are adequately suited to their health objectives might lower the number of necessary diagnostic tests and the readmission rate. It can help physicians by automatically identifying potential issues and alerting medical staff [22]. Additionally, they would lessen the likelihood of misdiagnoses and medical malpractice claims, which can add to costs.

AI applications can deal with the enormous amounts of data generated in the medical field and discover valuable knowledge that would otherwise be hidden in big medical data. Healthcare stakeholders may use AI-based computational tools to harness the power of data to review historical data, anticipate prospective outcomes, and identify the optimal actions for the current context. As a result, AI is becoming more essential to healthcare stakeholders in decision-making [23]. When putting privacy protection measures in place inside a specific system, this service represents a possible privacy breach that must be considered. End users are now more concerned than ever with the privacy of their health data due to increasing awareness among them [24]. New types of cyber-attack will be made possible by advances in AI. These attacks may use AI systems to do specific tasks more effectively than humans could or exploit flaws in AI systems that humans cannot control.

Additionally, AI systems regulate elements of malware and robot behavior that are impossible for humans to hold [25] manually. In the past, several security measures were put out to protect the transmission of patient data to hospitals [26,27,28,29]. However, the high cost and lengthy process prevent the best option from being implemented. Therefore, this research proposes a new cost-effective security algorithm for an intelligent hospital management system for COVID-19 data transmission. The significant contribution of this research is summarized as follows:

▪ Gather the IoT-sensed data of COVID patients from different remote areas.
▪ Apply the LRO-based serpent (S) encryption algorithm to secure data transmission.
▪ The asymmetric hash signature function is validated for key validations from the sender and receiver.
▪ Investigate the effectiveness of the proposed system using various parameter metrics.

The remaining parts of the article are arranged as follows. The access control model’s benefits and shortcomings are discussed in Section 2 of the literature review. Section 3 covers our proposed system. Section 4 of the concept discusses the performance evaluation of the algorithms. Section 5 concludes the work by providing recommendations for more research.

## 2. Related Work

In this section, we review the most recent research and compare the options that are currently available for security and anonymity in smart healthcare systems. As new devices proliferate, they often integrate the Internet of Things (IoT), generating and exchanging a massive quantity of data in the process. As a result, providing protection in an IoT setting is more difficult than expected. Properties such as secrecy, integrity, authorization, privacy, permission, and availability must all be ensured in order to ensure security in the IoT. Following is a summary of specific recent articles related to this research: Thilagam, K. et al. [30] offered IoT-based deep learning techniques based on privacy protection and a data analytics system. The health-related data are examined in the cloud using a convolutional neural network (CNN), free of user privacy data. As a result, a safe access control component is introduced for the IoT–Healthcare system based on user attributes. Furthermore, a higher user count enables an accuracy of about 98%. Experimental research shows that the suggested solution is reliable and efficient in terms of little privacy leakage and good data integrity.

Ali, Aitizaz et al. [31] created a novel deep-learning strategy-based secure searchable blockchain that functions as a distributed database and uses homomorphic encryption to allow users to access data safely via search. Using an IoT dataset, this study evaluated and compared the recommended access control mechanisms to reference models. The hyper ledger tool’s smart contracts implement the suggested algorithms. Compared to reference models, our proposed method considerably enhances security, privacy, and user behavior tracking, leading to a more effective blockchain-based IoT system.

Deep learning (DL) methods were combined with authorized blockchain and intelligent contracts by Kumar, Randhir et al. [32] to create the unique, safe, and effective data-sharing model PBDL. To be more precise, PBDL has a blockchain-based system to register, authenticate (using zero-knowledge evidence), and verify the communicating parties before employing an innovative contract-based agreement method. The healthcare data are encoded or transformed into a new format using stacked sparse variational autoencoding (SSVAE) in this technique. In addition, the attack detection mechanism is identified and enhanced using self-attention linked bidirectional long short-term memory (SA-BiLSTM).

Kute, Shruti Suhas et al. [33] provided a study of cutting-edge research involving the IoT in healthcare, particularly on obesity, overweight, and persistent degenerative illnesses. Secrecy, integrity, authentication, access, trust, validation, information management, and storage and availability issues must be resolved for IoT in real-world applications. A description of the security, privacy, and trust problems in IoT-based machine learning depending on healthcare systems is also provided in this study.

Using a hybrid deep neural network system, Ali, Aitizaz et al. [34] proposed a new group theory (GT) that depended on the binary spring search (BSS) technique. The blockchain was presented as a distributed database to guarantee secure tracking and keyword-based access to the dataset. The proposed methodology also offered a secure critical revocation method, and various policies were updated accordingly. The security of patient healthcare information access systems incorporating blockchain and a confidence chain addressed the efficiency and safety difficulties in the existing schemes for exchanging both forms of digital healthcare data.

One such IoT and cloud computing application was the topic of a study by Anuradha, M. et al. [35]. This work’s primary goal was to develop a cancer prediction system utilizing the Internet of Things after extracting the specifics of blood results to determine whether they were normal or abnormal. Additionally, the blood results of cancer patients were encrypted and stored in the cloud for easy Internet access by doctors and nurses who needed to handle patient data discreetly. This focused on improving the calculations and processing in the healthcare industry. To offer authentication and security when dealing with patients with cancer, encryption and decryption were performed using the AES method. 

Initial emphasis was placed on the fundamental security requirements for a Body Sensor Network (BSN)-based contemporary healthcare system. As a result, BSN-Care was proposed, a successful IoT-based healthcare system that enabled BSN to effectively meet these requirements Satyanarayan et al. [36].

The Authentication, Authorization, and Audit Logs (AAA) services were achieved by FBASHI, a system built on blockchain technology and fuzzy logic Zulkifl, Z. and Khan et al. [37]. This work provided a heuristic method for conducting driven flexible security, offering AAA services for medical care IoTs and networks based on the blockchain. It also suggested an approach for action driven flexible security using fuzzy logic.

For IoT-enabled hospitals, a reciprocal authentication method that protects privacy was suggested by Das, S. and Namasudra in order to accomplish quick and efficient network device verification [38]. This suggested authentication method was built using lightweight cryptographic primitives, such as XOR, combination, and hash operation, to accommodate the computing power of the IoT devices. The suggested strategy could block unwanted devices from accessing healthcare networks by establishing a safe connection between an approved device and a gateway.

The summary of related work is provided in Table 1. In all of these approaches, data protection and confidentiality are lessened. Additionally, all of the aforementioned techniques usually come lacking in terms of security efficiency and accuracy. The performance metrics are much less for evaluation and inaccurate for huge amounts of data. Consequently, this study suggests a novel efficient optimization-based security method for data transfer in an intelligent healthcare management system.

## 3. Proposed Methodology

**Figure 1:**
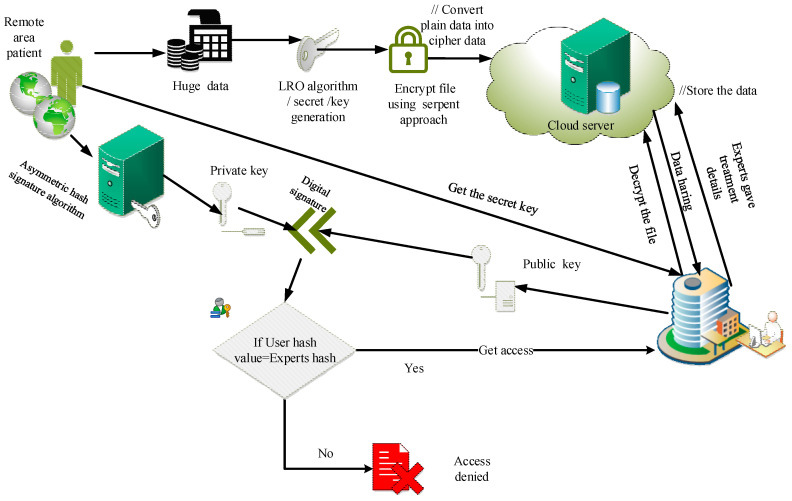
Proposed model of security system in smart hospital management.

The proposed design of security management in smart healthcare management is illustrated in Figure 1. The COVID data were collected locally and globally by IoT-based sensors, which was helpful for electronic medical records administration. The serpent (S) encryption technique based on LRO to protect data transfer from sensed data was applied. The LRO algorithm created the secure key for the serpent algorithm. The wearable IoT device stored its acquired data on a cloud server and was open to hacker attacks and privacy violations from unauthorized users. The asymmetric hash signature function was validated in the intelligent healthcare management system for critical validations from the sender and receiver. If both perform the same position, only the secret key was sent to the recipient, who may then use it to decode the data. A similar process was used for hospital-based medical professionals.

### 3.1. Lionized Remora Optimization

The LRO is the combination of the lion and remora optimization functions. The functions of both systems are hybrid to form an improved approach. The parameter in the problem $k_{i}=(k_{i1},k_{i2}\ldots k_{in})$, where i is the number of the secret key, n is the dimension in the search space of the secret key and represents the current point, and the possible solution in the suggested LRO technique is meant to be a secret key. The ideal algorithmic solution is symbolized by $k_{best}$, and how the goal in biological actions is represented is $k_{best}=(k_{1}^{*},k_{2}^{*},\ldots ,k_{n}^{*})$. An algorithm should have a fitness function for each probable solution. It might be worded such as this $f(k_{i})=f(k_{i1},k_{i2}\ldots k_{in})$. The equivalent formula for calculating the fitness function’s value is f. Using Equation (1), the algorithm saves the best fitness value associated with the best secret key location.

(1) $$f(k_{best})=f(k_{1}^{*},k_{2}^{*},\ldots ,k_{n}^{*})$$

Furthermore, the secret key, scattered around the search area, is the key to finding a solution. Other marine life or ships are just aids in the secret key’s mechanism for updating locations; they are not the method itself. These technologies allow the secret key to find the appropriate place in the neighborhood.

**Key Exploration**: One may imagine the target point updating simultaneously with the secret key connected to it. The position update formula was modified to offer the following equations based on the novel idea underlying this method:

(2) $$k_{i}^{t+1}=k_{best}^{t}-\left(r(0,1)*\left(\frac{k_{best}^{t}+k_{r}^{t}}{2}\right)-k_{r}^{t}\right)$$

In the above equation, *T* denotes the total number of iterations while, and t denotes the number of iterations that have already occurred. $k_{rand}$ designates a random location. Elite decides when to begin the upgrading in the traditionally preferred location of the secret key. Secret keys are added randomly to make it possible to explore the search area. Whether an animal has devoured prey or its current fitness level is higher than the previous generation largely determines which secret key to use for which host. Actually, “Experience attack” is utilized to calculate the value of the current fitness function. Similar to how experience accumulates over time, the tuyu must frequently take a little step around the host to determine whether it is necessary to move hosts. When the notions mentioned earlier are modeled, the formula is as follows:

(3) $$k_{a}=k_{i}^{t}+\left(k_{i}^{t}-k_{p}\right)*r$$

where $k_{p}$ denotes the perspective of the previous generation, which may be viewed as a type of experience, and $k_{a}$ suggests a reluctant action. The decision to utilize the r is made because when the secret key moves only so aggressively; it can be perceived as a “small global” movement. This mechanism, which has seen a more comprehensive range of evolution, may successfully depart from the local optimal while considering predictability. A decision-making stage is then required, after which the secret key randomly chooses whether or not to repair the host. The comparison of the fitness function values between the suggested solution $f(k_{i}^{t})$ and the current solution $f(k_{a})$ are used to evaluate this algorithmic phase. The condition for the point

(4) $$k_{i}^{t}=\{\begin{array}{c} \begin{array}{cc} r((2\times h-k_{i}^{t}),h), & (2\times h-k_{i}^{t})<h \end{array}\\ \begin{array}{cc} r(h,(2\times h-k_{i}^{t})), & (2\times h-k_{i}^{t})>h \end{array} \end{array}$$

where h is the current weight of the key, r is the random number, and $k_{i}^{t}$ is the new weight of the key. The center value of the key point is evaluated using Equation (5)

(5) $$k_{ic}^{t}=\{\begin{array}{c} \begin{array}{cc} r(k_{i}^{t},h), & k_{i}^{t}<h \end{array}\\ \begin{array}{cc} r(h,k_{i}^{t}), & k_{i}^{t}>h \end{array} \end{array}$$

The successive rate of this execution for best fitness is achieved using Equation (6)

(6) $$S(e,t,k)=\{\begin{array}{c} \begin{array}{cc} 1 & f_{e,k}^{t}<f_{e,k}^{t-1} \end{array}\\ \begin{array}{cc} 0 & f_{e,k}^{t}=f_{e,k}^{t-1} \end{array} \end{array}$$

When attempting to solve the minimum problem, for instance, if the fitness function value obtained from the LRO is less than the value obtained by the current solution,

(7) $$f(F_{i}^{t})<f(F_{a})$$

The secret key for the local optimum chooses a unique feeding approach. Host selection will resume if the suggested solution’s fitness function value exceeds the current solution’s value.

**Key Exploitation**: The equation for changing the location of the whale’s secret key was deleted. As illustrated below:

(8) $$k_{i+1}=g*e^{\delta }*\cos(2\pi \delta )+k_{i}$$

(9) δ=r(0,1)∗(x−1)+1

(10) $$x=-\left(1+\frac{t}{T}\right)$$

(11) $$g=\left|k_{best}-k_{i}\right|$$

The positions of a secret key attached to a whale can be taken for granted in a larger solution space. Here, g is the distance between the attacker and is the best choice at the moment; δ is a random value between [1, 1] and [2, 1] and shrinks exponentially after that. Host feeding is another step in the exploitation process. Now, the ideal solution may be compressed to the location space of the host. Small actions performed on or close to the host are described mathematically as:

(12) $$k_{i}^{t}=k_{i}^{t}+v$$

(13) $$v=d*(k_{i}^{t}-e*k_{best})$$

(14) d=2∗v∗r(0,1)−y

(15) $$y=2*\left(1-\frac{t}{T}\right)$$

**Figure 2:**
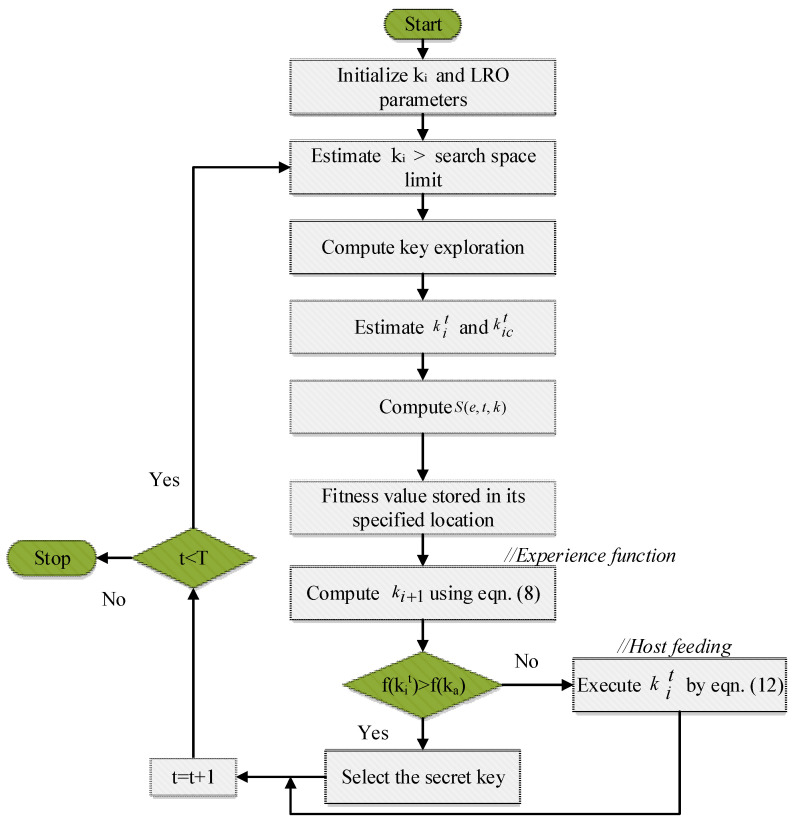
Flowchart of the LRO algorithm.

In this instance, v was used to denote a little movement associated with the volume space of the host and secret key. The position of the secret key was to differentiate between the functions of the host and secret key in the solution space, and the position of the secret key was constrained e using a secret key factor. If the host had a volume of 1, the volume of the secret key was about a portion of the host’s volume. Once the ideal answer had been found, the function ceased; otherwise, it continued for the following iteration. Figure 2 provides the LRO algorithm flowchart.

### 3.2. Serpent Security Strategy

We presented a serpent model, an extremely efficient block cipher architecture that is also quite conservative. It employs S-boxes such as those of the Data Encryption Standard (DES) in a novel form that permits a faster avalanche, a more effective bit slice execution, and a simple analysis that lets us prove it’s secure against all known types of attacks. The serpent has a block size of 128 bits and uses a 32-round network with four 32-bit words. Bitstreams are used to encode every value that is utilized in the encryption. In a single 32-bit word, the bit pointers are numbered from 0 to bit 31, from 0 to bit 127 for 128-bit blocks, from 0 to bit 255 for 256-bit keys, and so on. All values are written in the little for internal calculation, where word 0 is the least relevant, word n is the most significant word, and bit 0 is the least pertinent bit of word n. Each block is represented externally as a simple 128-bit hex integer. The plaintext is transformed into the first intermediate data, $C_{o}=Q$, and then the 32 rounds are used, each of which has three operations j∈{1,2,…31}. 

Key Collaboration: A 128-bit subkey $k_{j}$ is exclusive OR’ed with the present intermediate node $C_{j}$ at each round.

S-Boxes: Four 32-bit words make up the 128-bit input + key combination. These four words are put through the S-box, implemented as a series of logical operations, producing four output words as a consequence. As a result, the CPU is used to run all 32 duplicates of the S-box at once, producing using Equation (16)

(16) $$S_{j}\left(C_{j}⊕k_{j}\right)$$

Linear Transformation: Each of the output words’ 32 bits is linearly mixed, by

(17) $$Y_{0},Y_{1},Y_{2},Y_{3}:=S_{j}\left(C_{j}⊕k_{j}\right)$$

(18) $$When\{\begin{array}{c} Y_{0}:=Y_{0}<<<13\\ Y_{2}:=Y_{2}<<<3\\ Y_{1}:=Y_{1}⊕Y_{0}⊕Y_{2}\\ Y_{3}:=Y_{3}⊕Y_{2}⊕(Y_{2}<<3)\\ Y_{1}:=Y_{1}<<<1\\ Y_{3}:=Y_{3}<<<7\\ Y_{0}:=Y_{0}⊕Y_{1}⊕Y_{3}\\ Y_{2}:=Y_{2}⊕Y_{3}⊕(Y_{1}<<7)\\ Y_{0}:=Y_{0}<<<5\\ Y_{2}:=Y_{2}<<<22\\ C_{j+1}:=Y_{0},Y_{1},Y_{2},Y_{3} \end{array}$$

where << stands for shift and <<< for rotation. This linear transformation is substituted in the last round by an extra key mixing:

(19) $$C_{32}:=S_{7}\left(C_{31}⊕K_{31}\right)⊕K_{32}$$

To enhance the avalanche effect, the linear transformation was used in the first place $IP=\left(C_{j}\right)={\hat{C}}_{j}$ and $IP=\left(k_{j}\right)={\hat{k}}_{j}$. Since the different pairs of 0 through 1, 3, 5, 7, and 13 modulo 32 have only one ordinary member, the S-boxes receive the property that a 1-bit transformation will result in two variables to the output bits. A 1-bit change will result in maximum bit changes after two rounds and beyond. After three rounds, each plaintext bit and round key bit impact all the data bits. It is still assured that each key bit impacts each information bit for six cycles, even if an adversary selects certain subkeys and proceeds backward. The second argument is that it is straightforward and requires the fewest pipeline delays possible on a modern processor. The third reason is that we could put constraints on the probability of the differential and linear features by analyzing them using the block cipher analysis algorithms we built. These constraints demonstrate how this option is appropriate for this research.

**Figure 3:**
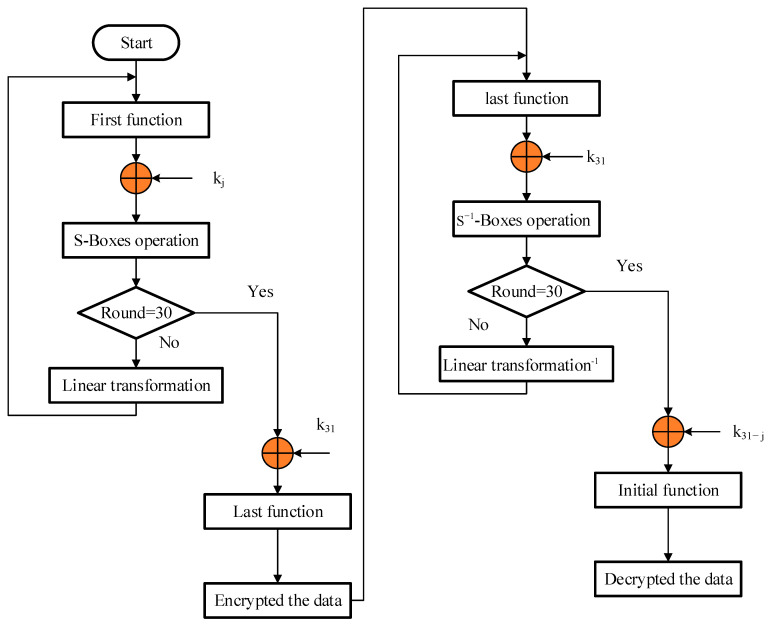
Serpent data security system.

Decryption: Inverse S-boxes, an inverse linear transformation, and the subkeys’ reversed order are all necessary for decryption, which sets it apart from encryption. The flowchart of the serpent security approach is illustrated in Figure 3.

### 3.3. Asymmetric Hash Signature

**Figure 4:**
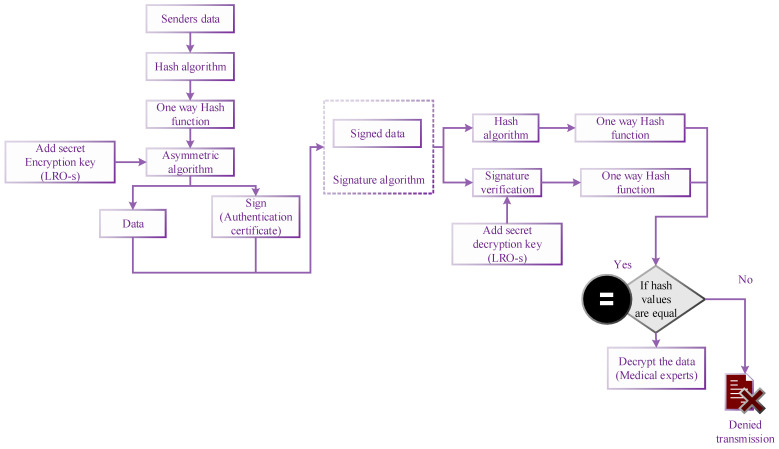
Flowchart of proposed Asymmetric Hash Signature.

The security of this system is improved more than by the encrypted hash function. If the hash function of both senders and the medical experts are the same, then only they can access the data. A key generation technique randomly chooses one private key from a list of potential private keys. The LRO algorithm produces the private key and a matching public key. A signature-producing signing method generates a signature from data and a private key. The process for validating signatures either accepts or denies the data’s claim to authenticity depending on the data, public key, and sign. The signing algorithms establish a one-way hash of the digital data that has to be signed to create a digital signature. The hash value is, subsequently, encrypted by the signing method using the private key. The signature consists of this encrypted hash and other details such as the hashing technique. Finally, the data are transmitted to the verifier with this signature attached. Because a hash function may transform any random input into a much smaller fixed-length result, it is preferable to encrypt the hash rather than the entire message or document. Therefore, it will help to save time, since one must now sign a smaller hash value instead of a lengthy document, and hashing is considerably quicker than signing. The same hash function produces a hash value from the received data. The signature is legitimate if they are both equivalent; otherwise, it is illegitimate. The flowchart of the proposed Asymmetric Hash Signature is illustrated in Figure 4.

## 4. Result and Discussion

The proposed security framework was built on a Windows system equipped with an i5 CPU and 4 GB of RAM using the MATLAB 2019a program. In this research, COVID-19 data are used to transmit and validate the security of the system. The data are gathered from a database management system (DBMS), which was initially established in the 1960s. DBMSs often provide database–server capability. Several DBMSs (such as MySQL) only use the client–server paradigm for accessing databases, whereas some (such as SQLite) are designed to be used as integrated databases [39]. Patients can use the DBMS to see and compare different treatments for a specific patient’s clinical condition, allowing them to select a course of therapy that is also practical for their insurers. The patient’s health status will be recorded by a sensor in this health tracking device. However, the DBMS makes the data look complex. Moreover, several factors, such as encryption and decryption times, time complexity, energy use, cost-effectiveness, and others, were validated for the performance analysis of the suggested model compared to the existing approaches, and an optimization algorithm and a security algorithm were used to ensure that the data transmission process was carried out securely. 

### 4.1. Performance Analysis

The proposed method was compared with the different conventional methods such as CNN [23], homomorphic [24], PBDL [25], GT-BSS [27], and AES [28] in terms of encryption time, decryption time, resource optimization, execution time, delay, key generation, and so on.

### 4.2. Encryption Time

The time it takes to encrypt data from plain to cipher data is the encryption time.

(20) $$\mathrm{Encryption}\;\mathrm{time}=\frac{\mathrm{Overall}\;\mathrm{encrypted}\;\mathrm{plain}\;\mathrm{data}(\mathrm{kb})}{\;\mathrm{time}(\mathrm{ms})}$$

**Figure 5:**
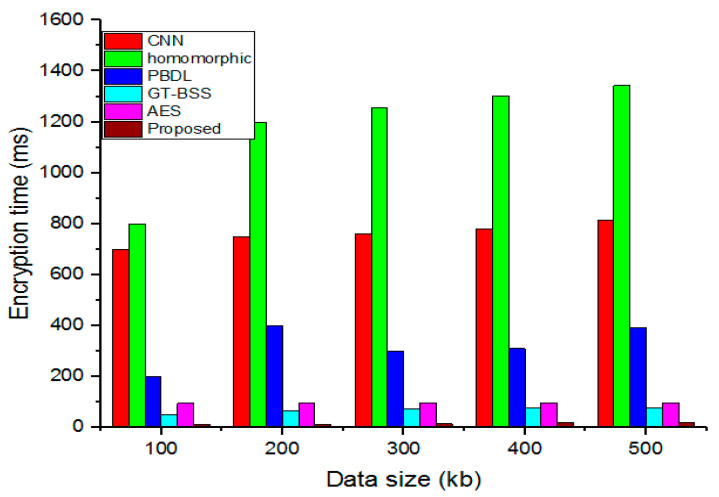
Analysis of variations in encryption time.

Figure 5 compares the projected time required for encryption to the current models. The encryption process takes longer as the data size increases. The presentation demonstrates that the suggested technique outperforms the standard methods for files of various sizes, including 100 kb, 200 kb, 300 kb, 400 kb, and 500 kb, due to the suggested model’s far shorter encryption time than the preceding approaches.

### 4.3. Decryption Time

The time it takes to decode data from cipher data to original data is referred to as decryption time.

(21) $$\mathrm{Decryption}\;\mathrm{time}=\frac{\mathrm{Overall}\;\mathrm{decrypted}\;\mathrm{cipher}\;\mathrm{data}(\mathrm{kb})}{\mathrm{time}(\mathrm{ms})}$$

**Figure 6:**
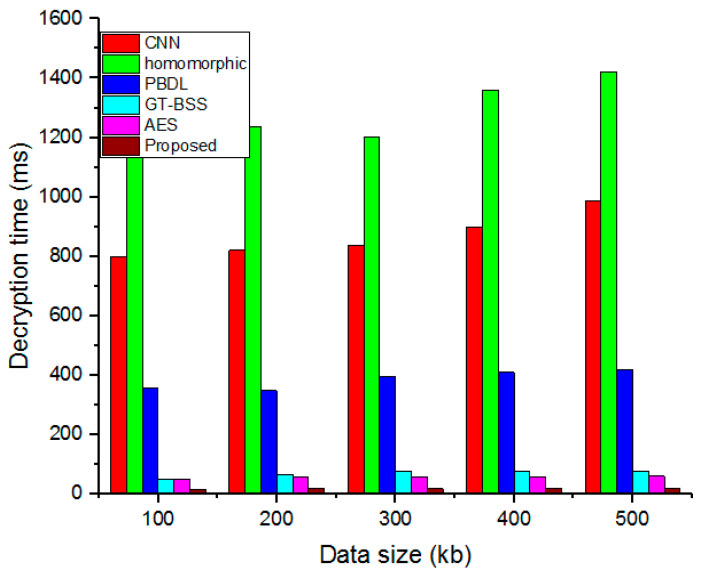
Analysis of differences in Decryption time.

The suggested time needed for decryption is compared to the present models in Figure 6. As data size grows, so does the amount of time required for decryption. The presentation demonstrates that the suggested technique outperforms the standard methods for files of various sizes, including 100 kb, 200 kb, 300 kb, 400 kb, and 500 kb. In addition, because it takes much less time to decode data than prior approaches, the suggested model offers several advantages.

### 4.4. Key Generation

**Figure 7:**
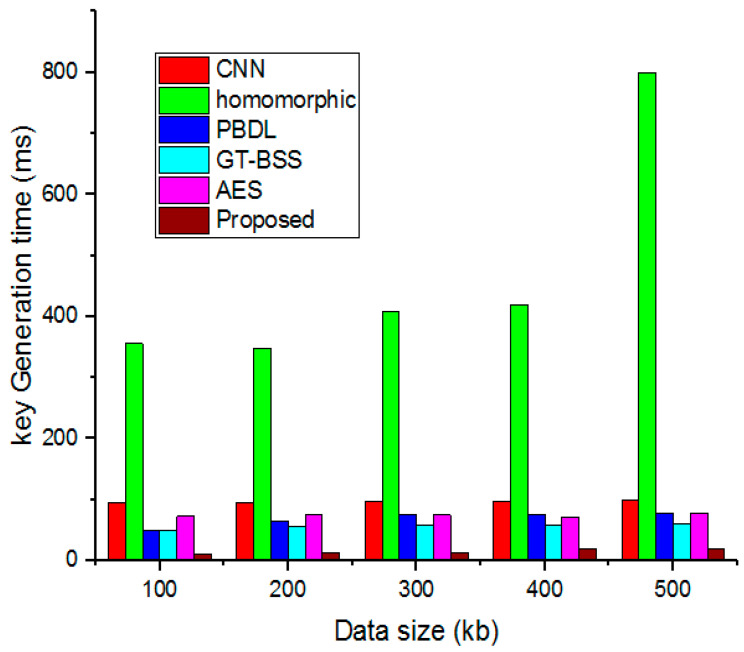
Comparative analysis of key generation.

Figure 7 compares the time required for key generation in the proposed security system with various values from previous models. Key generation is much quicker than conventional methods.

### 4.5. Key Size

**Figure 8:**
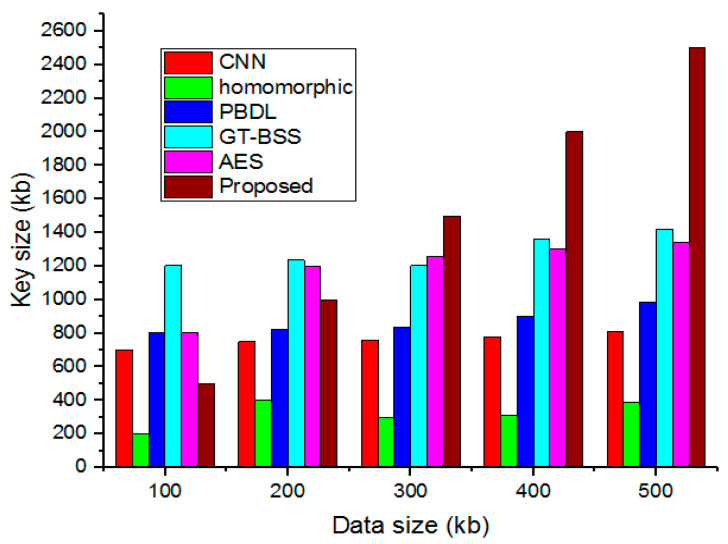
Comparative analyses of key sizes.

Keys regulate a cipher’s operation; only the correct key can decrypt a communication and reveal its plain text content. Since many encryption methods are based on or made publicly available, the system’s security, assuming no analytic attack, is only decided by how difficult it is to obtain the key. Therefore, estimating key size is crucial since it establishes the number of bits in a key that a security method will use. Figure 8 provides the comparative analysis for key size.

### 4.6. Confidential Rate

**Figure 9:**
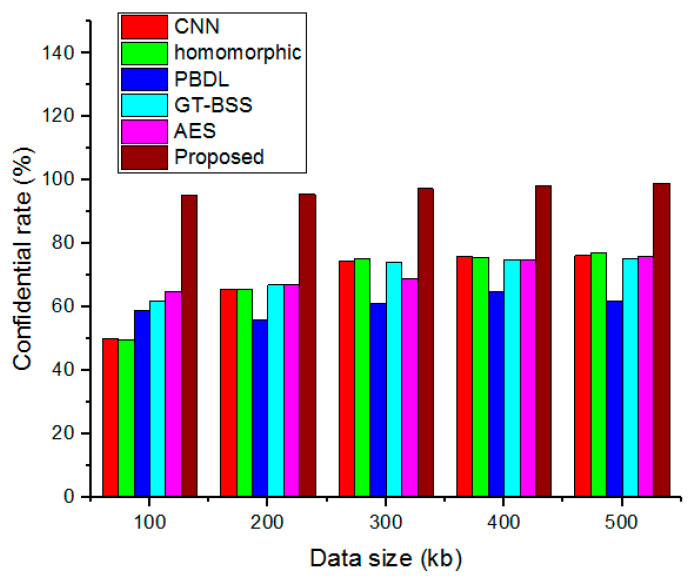
Performance analysis of the confidential rate.

The confidential rate is based on the differential between data that is sent and received during a data transfer. Figure 9 compares the performance analysis of the suggested confidential rate with the traditional approaches. The proposed system’s validation of the confidential rate is estimated for various file sizes. The conventional method has, however, become much less secretive over time. The results demonstrate how well the suggested model’s security function works.

### 4.7. Resource Optimization

**Figure 10:**
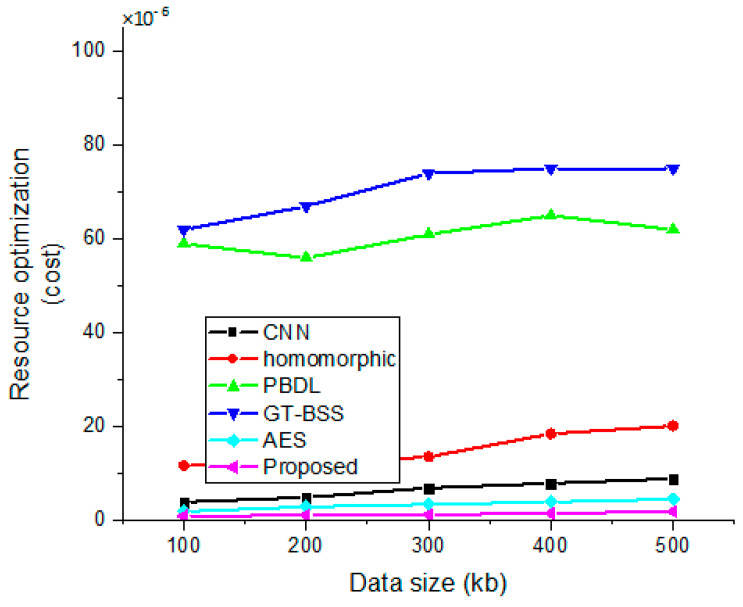
Correlation between data size and resource optimization.

The transfer between data size and resource optimization is shown in Figure 10. It has been shown that the cost grows linearly with data size. Compared to the preceding models, the cost is optimized to an impressive degree.

### 4.8. Execution Time and Delay

**Figure 11:**
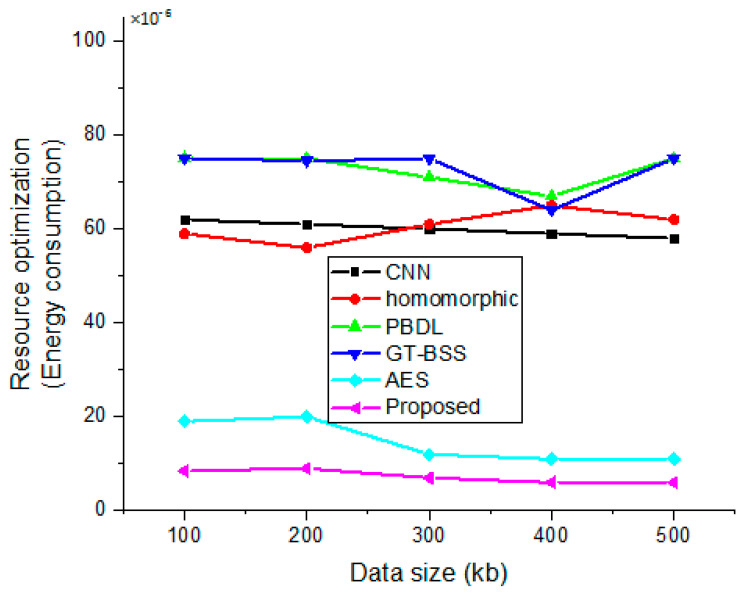
Correlation between data size and energy consumption optimization.

The relationship between data size and resource optimization is depicted in Figure 11, and it is deduced that energy consumption decreases as the quantity of data increases, but the charge drain increases. Additionally, it has been noted that resources are cost-effectively optimized. 

**Figure 12:**
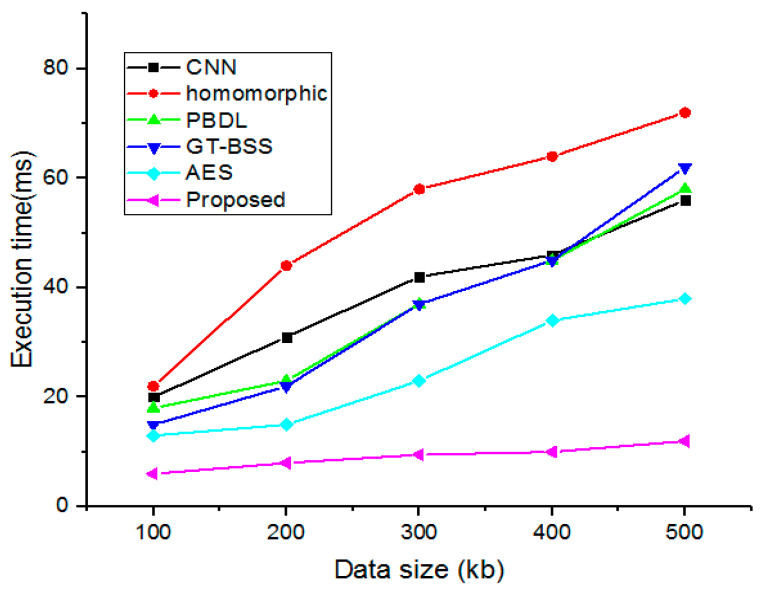
Execution time for complete data size.

**Figure 13:**
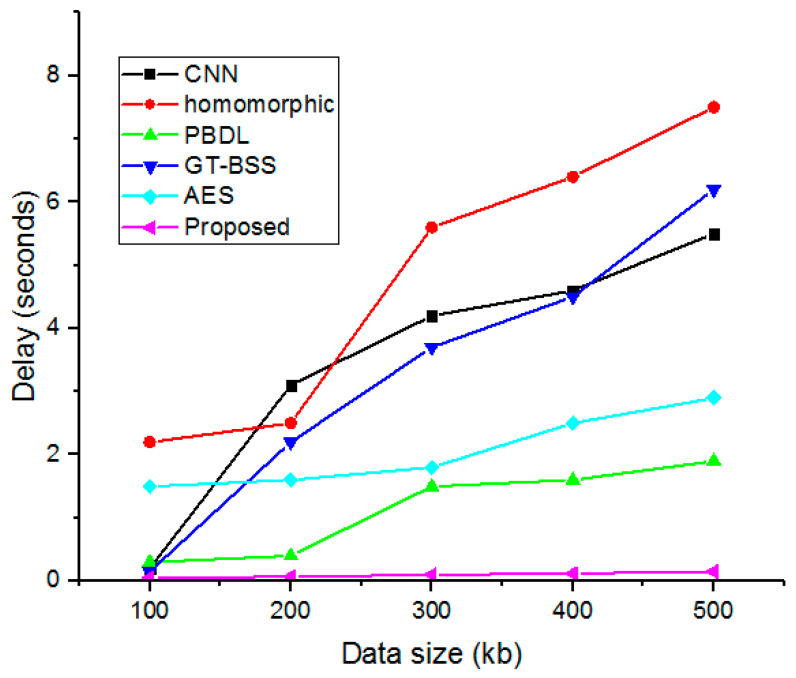
Delay estimation for complete data size.

Execution time and average delay are the two crucial factors when assessing the implemented model. However, data transmission computation and average delay for all employed smart hospital management data security are nearly consistent, as illustrated in Figure 12 and Figure 13. The suggested methodology is, therefore, highly scalable regarding the volume of data and the number of data transmissions carried out. Furthermore, in a smart hospital framework, our proposed solution provides the safe storage and accessibility of dispersed medical records. Thus, the analysis shows the adequate performance of the proposed security system over the earlier methods.

## 5. Conclusions

The use of cost-effective security in an intelligent hospital healthcare system was the primary focus of this research study. Here, remote patients’ COVID-19 information was gathered and processed via the IoT. As a result, sensitive patient data were encrypted using the LRO-S method and then saved in a cloud environment. Then, an asymmetric hash signature method was used to strengthen the security mechanism. Increasing the security and flexibility of medical professionals’ access to patient information stored in the cloud was the key focus of this research project. According to the testing findings, the secret keys created from upgraded participants were sufficiently random and one-of-a-kind to protect the IoT in smart hospital management systems. The effects of the suggested approach demonstrated a decrease in encryption and decryption times and an increase in confidentiality rates. The proposed healthcare framework used less energy, money, and processing time than the techniques examined. This study ensured the safe communication of medical information between patients and doctors while reducing healthcare costs. Future research will use deep learning technology to predict and protect against numerous cancers, including breast, blood, lung, skin, and other types.

## Figures and Tables

**Table 1:** The summary of related works.

Reference	Methods	Systems	Key Results	Advantages	Limitations
Thilagam, K. et al. [30]	CNN	Private healthcare data	Accuracy, Recall, F1-score, Precision, False Alarm Rate, and Missed Detection Rate.	Data integrity and privacy leaks are both minimal.	High time and cost consumption.
Ali, Aitizaz et al. [31]	homomorphic encryption	Digital healthcare	Throughput, encryption time, decryption time, latency, and computational cost.	Gives consumers more flexibility.	The loss function is high.
Kumar, Randhir et al. [32]	PBDL (SSVAE and SA-BiLSTM)	Industrial healthcare	Transmission efficiency, encryption and decryption time, accuracy, and loss.	Secured authenticated data transmission and attack detection.	More extended training period and slower prototype.
Kute, Shruti Suhas et al. [33]	Machine learning	IoT-based healthcare	Accuracy, loss, and confidential rate.	Effective validation is achieved for different sickness.	Need to address real-time challenges.
Ali, Aitizaz et al. [34]	GT-BSS	Digital healthcare	Throughput, encryption time, decryption time, latency, and computational cost.	Limits security problems to patient data.	Very low test accuracy.
Anuradha, M. et al. [35]	AES	Cancer prediction system	Cost and time.	High-security function.	A small amount of data is considered for validation.
Satyanarayana, T.V.V. et al. [36]	BSN	Medical system	CPU cycles and execution time.	Security needs are effectively solved.	Very few metrics are validated for the performance evaluation.
Zulkifl, Z., Khan et al. [37]	FBASHI	Hospital department	Latency and throughput.	Different kinds of attacks are evaluated.	Lack of evaluation metrics.
Das, S. and Namasudra [38].	Lightweight cryptographic primitives	Healthcare center	Computation cost and execution time.	Feasible for lightweight and low resource IoT gadgets.	Only applicable for low resource IoT gadgets and systems. Additionally, less data.

## Data Availability

The datasets that were used in this study are available online on the following link IoT Healthcare Security Dataset https://www.kaggle.com/datasets/faisalmalik/iot-healthcare-security-dataset accessed on 15 November 2022.
